# Early evidence for historical overfishing in the Gulf of Mexico

**DOI:** 10.1126/sciadv.abh2525

**Published:** 2021-08-04

**Authors:** Eric J. Guiry, Jonathan R. Kennedy, Martin T. O’Connell, D. Ryan Gray, Christopher Grant, Paul Szpak

**Affiliations:** 1 School of Archaeology and Ancient History, University of Leicester, Mayor’s Walk, Leicester LE1 7RH, UK.; 2 Department of Anthropology, Trent University, 1600 West Bank Dr., Peterborough, ON K9L 0G2, Canada.; 3 Department of Anthropology, University of New Orleans, Milneburg Hall 352, 2000 Lakeshore Dr., New Orleans, LA 70148, USA.; 4 Department of Earth and Environmental Sciences, University of New Orleans, 1065 Geology and Psychology Building, 2000 Lakeshore Drive, New Orleans, LA 70148, USA.; 5 Department of Anthropology, University of Chicago, 1126 East 59th Street, Chicago, IL 60637, USA.

## Abstract

Fisheries encompass complex interplays between social, economic, and environmental factors, but limitations on historical fisheries data can hamper efforts to identify and contextualize the long-term spatiotemporal patterns that shape them. We integrate 2500 years of stable isotope (δ^34^S, δ^13^C, and δ^15^N) and zooarchaeological evidence from Gulf of Mexico fisheries to assess cultural, demographic, and technological changes affecting sheepshead (*Archosargus probatocephalus*) populations and fishing practices in Louisiana, USA. Concurrent with human population growth, average sizes of sheepshead caught decreased from the 1720s to 1830s. The size of fish caught after the 1830s increased to pre-1720 levels at the same time that isotopic compositions of fish bone collagen show that fish were being caught from a more diverse range of ecosystems, including distant seagrass beds. Our findings provide the first evidence for large-scale depressions of historical sheepshead populations and the processes driving them, including rapid human population growth and sustained harvesting pressure.

## Teaser

Early overfishing in the Gulf of Mexico highlights vulnerability of one of the world’s most productive marine fishery areas.

## INTRODUCTION

Of the global challenges facing our oceans, overfishing is the most impactful on both marine biota and the human societies that depend on them (*1*). It is widely recognized that, through technological advances over the last several decades, humans have been able to extract tremendous quantities of marine resources from oceans with relatively little knowledge of how these activities affect and change potentially sensitive ecosystems (*2*, *3*). Although the longer-term nature of historical impacts has been explored in some detail for European and other fisheries (*4*), the profound and yet often totally unrecorded historical impacts on marine ecosystems in many other regions of the world remain understudied, thereby limiting the potential for a global perspective on historical trends in human impacts on fisheries (*5*, *6*). With growing demands for seafood, compounded by escalating climate and food security uncertainties, overfishing is one of the greatest worldwide challenges for society this century, requiring substantial attention from academic, citizen, and government sectors (*1*, *7*).

Greater recognition of these issues is driving interest in improving conservation outcomes through the development of more nuanced management strategies that take into account the dynamic interactions among both ecosystems and human societies (*8*). In this vein, there is a growing awareness of not only the deep connections between the socioeconomic and symbolic dimensions of human marine resource use and the health and viability of marine ecosystems but also the long-term nature of these human-environment relationships [e.g., (*9*)]. Building more detailed retrospectives on the long-term evolution of human societies and marine fish communities can help contextualize both organism- and ecosystem-level responses to changing environmental conditions and fisheries practices as well as fishers’ responses to these changes, providing insights that can be valuable for improving conservation policy and guiding future environmental restoration efforts [e.g., (*10*–*12*)]. A key obstacle for this approach, however, is that in many regions of the world’s oceans detailed scientific observation of marine ecosystems, and fisheries impacts, typically only began in the 20th century, long after many ocean environments were heavily affected through the advent of industrialized and earlier large-scale fishing technologies (*13*). For this reason, in most cases, baselines for marine ecosystems reflect already profoundly altered environments that may not provide realistic targets for developing conservation management strategies (*5*, *14*). In recognizing the deeper antiquity of ecologically meaningful human impacts on aquatic environments, we can use longer-term retrospective evidence from archaeological and historical sources to search for clues about where, how, and why overfishing and other anthropogenic ecosystem changes have occurred in the past to contextualize and better chart new perspectives for the future.

Fishers respond to changes in fisheries in a variety of ways, including migration to new fishing grounds, diversification in the range of species targeted, and intensification of fishing effort for preferred species [e.g., (*15*)]. These trends have been observed in modern and historic fishing communities alike (*16*, *17*). Together, they are often associated with “fishing down the food web,” a hallmark of the global overfishing crisis in which fisheries sequentially remove progressively lower trophic level taxa as higher value species are fished out, becoming too scarce to remain economically viable (*18*). This phenomenon can drive ecosystem-wide changes resulting from successive population crashes, as industrial-scale fishers sequentially shift their attention among different species (*19*). For this reason, while much attention is put on protecting and restoring overfished species, to mitigate this broader process, it is equally critical that attention is also given to those taxa that have traditionally been overlooked in favor of larger and more valued fish but that are likely to come under increasing pressure (*20*).

Sheepshead (*Archosargus probatocephalus*), a marine fish cosmopolitan to coastal eastern North America, is emblematic of the fishing down the food web phenomenon in the Atlantic United States and Gulf of Mexico. Sheepshead are highly adaptable, allowing them to thrive in a wide range of coastal habitats, from freshwater to salt water. While sheepshead have always been fished by communities living across this vast coastal region [e.g., (*21*)], they have never been the primary focus of historically recorded fisheries (*22*). Despite culinary properties (sweet flavored meat with a flaky, tender texture) that are similar to more desirable and intensively fished species, the smaller size, lower meat yield, and relative difficulty in filleting of sheepshead compared to other commercially important taxa like red drum (*Sciaenops ocellatus*) have limited their popularity and modern commercial value (*23*). However, with sympatric stocks of more valuable fish becoming depleted in many areas of their range, sheepshead are increasingly coming under pressure. Annual sheepshead landings in the Gulf of Mexico, for instance, spiked to nearly 4 million pounds when trawlers began to target the species in the late 1980s and early 1990s, concomitant with a decline in abundance of red drum in the Gulf of Mexico and, eventually, a complete ban on commercial harvesting of the species across most of the Gulf (*24*, *25*). We might therefore look to sheepshead as a harbinger for the future of similar, traditionally overlooked but increasingly important fish species and how their overfishing could affect regional fisheries, ecologies, and future food security. In this context, conservation managers in key areas of the sheepshead’s range have expressed concern that fundamental aspects of the species’ ecology remain unstudied (*22*), making it difficult to develop realistic management targets in some areas.

In addition to key conservation questions, including basic details about the nature and location of spawning behavior (*22*), little information is available on how sheepshead populations have responded to increased fishing pressure in different habitats. Although historically smaller than more lucrative fisheries, in some places relatively large sheepshead fisheries have been sustained, and looking to these instances for historical clues may help us to better understand how the species responds to long-term changes in fisheries practices. Stable isotope analyses of archaeological bones provide a powerful tool for generating new insights into fish behavior and community structures in past and present fisheries. When integrated with other quantitative and qualitative archaeological data streams on catch size and composition, analyses have potential to address previously unanswerable questions about evolution and sensitivities of fisheries at a wide range of spatial and temporal scales (*11*). To contextualize current and future sheepshead fisheries, we undertake the first retrospective analyses of the impacts of fishing activities on sheepshead populations in and around New Orleans, home to what has historically been the largest of all sheepshead fisheries (*22*, *25*). Through osteological (morphology) and biogeochemical (stable isotope) analyses of fishbones from six archaeological sites in and around New Orleans spanning the past 2500 years (table S1), we provide new evidence for trends in sheepshead size and habitat to explore the ecological and cultural dimensions of previously undocumented overfishing events.

### Context

**Fig. 1:**
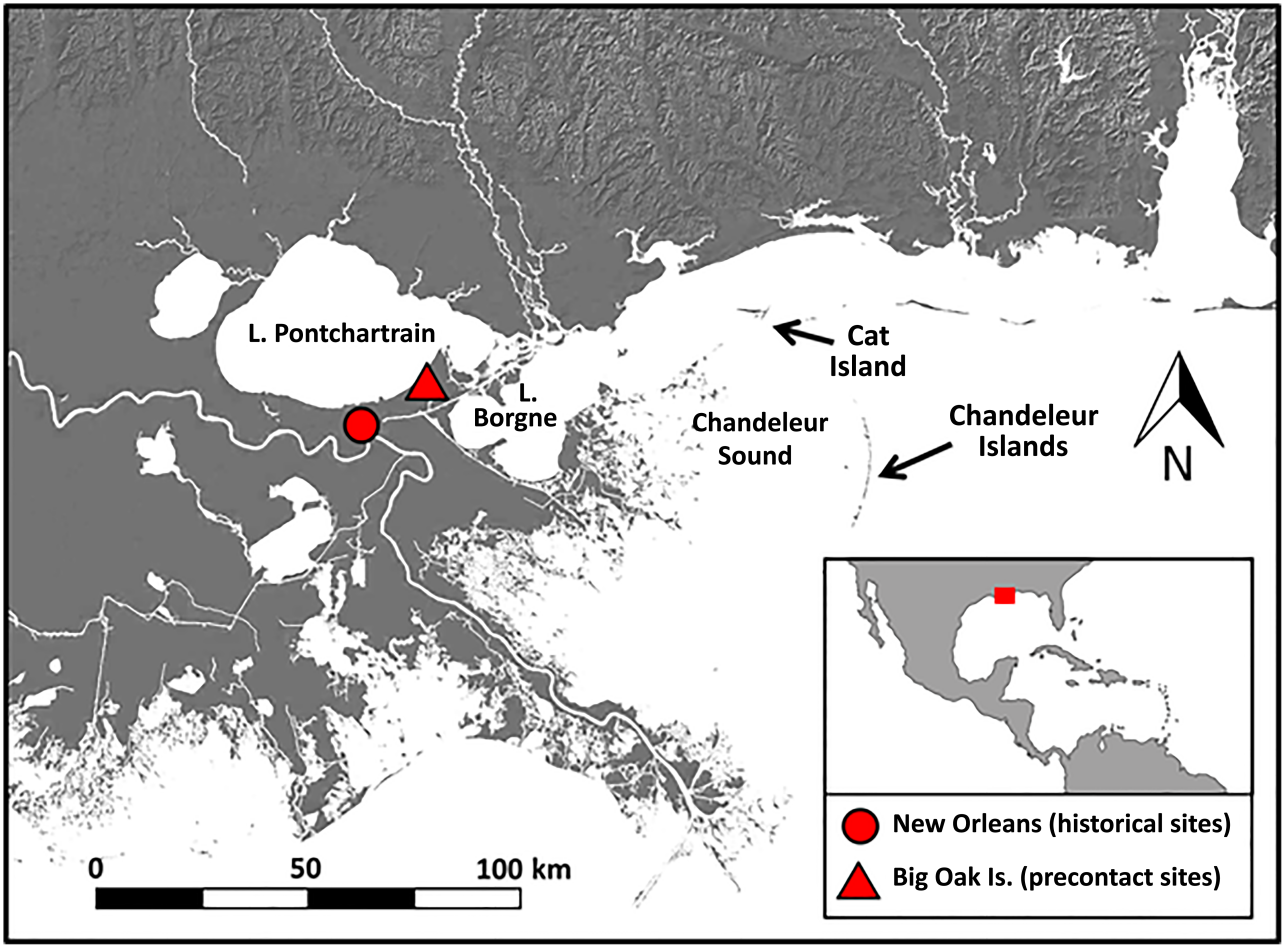
Map of study region. Inset shows study region (red rectangle) in broader context of the Gulf of Mexico.

Our study includes sheepshead bones recovered from five historical sites dating from ca. 1720 to 1910 CE and one precontact site dating from roughly 450 to 130 BCE (table S1) (*26*). Of these, 810 Royal Street (*27*) and Big Oak Island (*26*) have been previously reported on, while the remaining sites (1427 Ursulines Avenue, 626 Bourbon Street, 936 St. Peter Street, and Passebon Cottage) are the subject of ongoing analysis. The study area (Fig. 1) encompassing these sites contains a wide range of habitats used by sheepshead. This includes Lake Pontchartrain, a large brackish water estuary created through the alluvial action of the shifting Mississippi River. To the east is Lake Borgne, a higher-salinity estuary lagoon that ultimately leads to the Chandeleur Sound and the open waters of the Gulf of Mexico. Sheepshead populations not only move through Lake Pontchartrain and Lake Borgne but also inhabit coastal waters including those surrounding southeast Louisiana’s many barrier islands, including the Chandeleur Islands.

The earliest well-documented human settlement of the area was by the Early Woodland Tchefuncte people ca. 2500 years ago at archaeological sites such as Big Oak Island and Little Oak Island (Fig. 1), whose inhabitants made intensive use of aquatic resources, especially fish and brackish water *Rangia* clams (*26*). French colonists formally established the modern city of New Orleans in 1718, beginning three centuries of extensive environmental modifications to make the area suitable for urban development. These efforts included the successive construction of an urban center in what is now known as the French Quarter, development of suburban plantations and eventually neighborhoods, dredging of canals to drain swampland and provide navigable waterways, and levee building to control flooding from the Mississippi River. New Orleans saw rapid economic growth in the 19th century, and it became a destination for large numbers of immigrants in the early- to mid-19th centuries. Between 1830 and 1860, New Orleans’ urban population grew by 366%, in large part due to the arrival of large numbers of German, Irish, and other European immigrants (*28*). Historical and archaeological evidence indicate that seafood, especially fish, played a critical role in feeding New Orleans’ pre- and postcontact historic populations, with sheepshead being one of the most commonly identified species in postcontact archaeological assemblages throughout the city (*27*).

Small-scale, artisanal fishing accounted for most fish collection near New Orleans until the late 1700s (*27*, *29*). Bone fishhooks and possible net mesh gauges and net weights recovered from precontact archaeological sites in the area indicate that hook-and-line and net-based fishing methods were used by indigenous peoples before European arrival (*26*), and historical accounts indicate that similar methods were used by early colonial fishers near New Orleans (*30*). Beginning in 1763, the settlement of several thousand Canary Islanders (known as Isleños) near New Orleans during Spanish control of the city marked the development of professional fishing in the area (*31*). The Isleños found particular success fishing near and in Lake Borgne in what is modern St. Bernard Parish, and the regulation of fish markets in the late 1700s by Spanish authorities helped create a consistent outlet for fish supplied by these and other professional fishers (*32*). During the 1800s, additional immigrant communities including Croatians, Sicilians, Filipinos, and Chinese entered fishing and fishing-related industries, further enhancing commercial fishing operations in and around the city (*33*). Historical accounts from the late 1800s indicate that most fishers continued to rely on nets (especially seines and gillnets) to catch a range of fishes from coastal and inland waters, with small numbers of fishers using boats (smacks) to access fishing grounds (*34*). Last, rising consumer demand for fish in the late 1800s, alongside the completion of the New Orleans, Mobile, and Texas Railroad in 1871, led to the importation of reef fishes like snappers (Lutjanidae) and groupers (Epinephelinae) from Mobile Bay and Florida (*34*).

Beyond these historical trends described, a number of recent anthropogenic processes have directly affected fish populations in the study area and life in the Gulf of Mexico more broadly. For instance, an oxygen-poor dead zone driven by agricultural runoff from the Mississippi River now extends across the northern Gulf of Mexico; the wetlands of Louisiana and neighboring states are experiencing increasing erosion driven by the construction of numerous oil-related canals in the mid-20th century; and salinity changes driven by the construction of the Mississippi River–Gulf Outlet Canal have led to regime changes in nearby wetlands (*35*). These and other factors have contributed to instability and continued decline in modern Gulf of Mexico fisheries, creating a context in which understanding the historical foundations of modern fish populations and identifying previous fisheries impacts in the region are all the more important.

## RESULTS

**Fig. 2:**
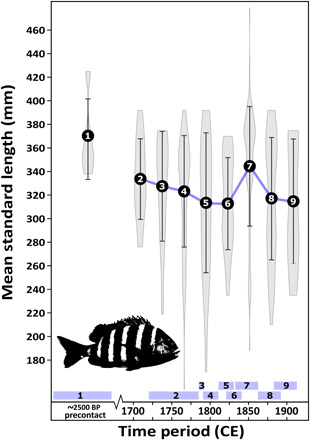
Sheepshead size change through time. Average estimated standard length derived from sheepshead bones from precontact and 18th- and 19th-century archaeological sites in New Orleans. Error bars show 1σ. Violin plots show size distribution. Time periods coded as follows: (1) Early Woodland Period [precontact, ca. 2500 before present (BP); *n* = 5], (2) 1720–1780 (*n* = 14), (3) 1789–1790 (*n* = 13), (4) 1790–1810 (*n* = 89), (5) 1820–1830 (*n* = 20), (6) 1830–1840 (*n* = 21), (7) 1840–1860 (*n* = 87), (8) 1870–1900 (*n* = 91), and (9) 1880–1910 (*n* = 13).

Results from osteometric analyses of 353 archaeological sheepshead bones (Fig. 2; data S1) show a systematic decrease in average standard length through time, indicating a long-term trend of declining size of sheepshead consumed in New Orleans. This overall pattern is punctuated by a spike in average standard length observed across 87 sheepshead specimens consumed at four separate sites in the 1840s to 1860s, which is, in turn, followed by a rapid decline in average size to pre-1840 numbers across an equally robust sample of sheepshead specimens dating to the latter decades of the 19th century. Interpretations of these trends are supported by statistical comparisons among neighboring temporal bin means, which showed no significant differences (*P* ≥ 0.050; full details in data S2) between sequential time periods (consistent with a gradual decrease) except for the 1840–1860 spike in fish size, which differs significantly from both the preceding (Mann-Whitney *U* = 496.5, *P* = 0.001) and succeeding (Mann-Whitney *U* = 2744, *P* < 0.001) time periods.

**Fig. 3:**
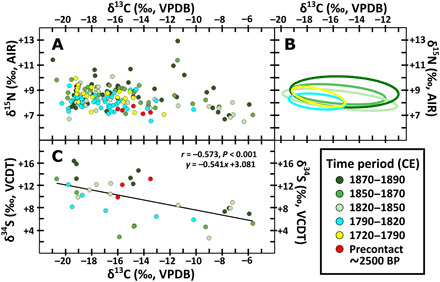
Sheepshead bone collagen stable isotope compositions by time period. (**A**) Stable carbon and nitrogen isotope compositions. (**B**) Comparison of standard ellipsis areas for δ^13^C and δ^15^N (precontact period not shown because of small sample size). (**C**) Stable carbon and sulfur isotope compositions.

Analyses of archaeological sheepshead specimens also produced a wide range of bone collagen stable isotope compositions (*n* = 182 for δ^13^C and δ^15^N, *n* = 34 for δ^34^S; Fig. 3, data S3, and table S2). Variation in δ^13^C (mean = −16.0 ± 3.4‰; range = −21.0 to −5.7‰), δ^15^N (mean = +8.4 ± 0.9‰; range = +6.5 to +12.9‰), and δ^34^S (mean = +9.6 ± 3.6‰; range = +2.7 to +16.4‰) indicates that sheepshead consumed in New Orleans were sourced from a broad range of brackish and marine habitats and are consistent with the species’ omnivorous feeding behavior and cosmopolitan habitat preferences (*36*). Substantial differences between the variation (quantified via SEAc) for δ^13^C and δ^15^N occur between sheepshead from earlier (SEAc range of 0.9 to 3.9 before 1820) and later time frames (SEAc range of 9.2 to 16.0 after 1820), resulting from a broadening isotopic niche of sheepshead consumed after 1820 (Fig. 3 and table S2). In particular, highly elevated δ^13^C values (ca. >−10‰) among later specimens indicate inclusion of fish that lived in ecosystems where primary production was heavily subsidized by saltmarshes or seagrasses [e.g., (*37*)]. As these sources of primary production also have unusually low δ^34^S for marine or coastal environments, their importance for sheepshead food webs is further supported by the strong negative correlation between δ^13^C and δ^34^S (*n* = 34, Pearson’s *r* = −0.573, *P* < 0.001) (Fig. 3) [e.g., (*38*)]. In that context, it is important to note the presence of saltmarsh habitat in local coastal areas (especially around Lakes Pontchartrain and Borgne), which would have been easily accessible during the 18th and early 19th centuries. For this reason, the fact that early fisheries in the New Orleans area do not appear to have incorporated fish with these distinctive isotopic compositions strongly suggests that they result from fish living in seagrass habitats that began to be harvested post-1820.

## DISCUSSION

Results highlight two parallel and complementary trends. First, osteological analyses (i.e., size estimation of archaeological sheepshead) reveal a clear pattern of decreasing sheepshead average standard lengths from the Early Woodland Period through the 1830s to 1840s, followed by an increase in average estimated standard length in the 1840s to 1860s, and, last, a return to consistently decreasing standard lengths from 1870 to 1910 (Fig. 2). Only five Early Woodland Period sheepshead samples were identified during the study, and given the low sample size, the high average estimated standard length of these fish may not be representative of the population from which they were caught; however, this period produced one of only three sheepshead samples in the study to be estimated to be from a fish longer than 420-mm standard length, at minimum suggesting that very large sheepshead were present in the study area during this period. The steady decrease in average estimated standard length from 1720 to the 1840s suggests long-term, negative impacts from fishing on sheepshead populations in the study area. Such decreases in average fish catch size are a well-documented response to overfishing and may result from use of size-selective gear [particularly gill netting, a common practice at the time; (*34*)] removing larger individuals, which, in turn, can promote genetic selection for earlier maturation at smaller sizes (*39*). During the 1840s to 1860s, however, average catch size sharply increases to the highest average estimated standard length of any postcontact period in the city, suggesting that fishers had begun to tap into new, previously un(der)fished sheepshead populations. However, the increased fish catch size was short-lived, as average estimated standard length dropped sharply in 1870–1900 and continued to decline through 1910, likely indicating overfishing of the newly targeted sheepshead populations.

Second, bone collagen stable isotope compositions of sheepshead landed during the precontact and early historical periods are consistent with fishing strategies focusing on local areas in Lake Pontchartrain, coastal bayous, and nearshore marine settings. Beginning in the 1820s, however, sheepshead stable isotope compositions indicate an expansion of the habits used by sheepshead landed in New Orleans. In particular, a large fraction of sheepshead that were landed after 1820 have highly elevated δ^13^C and low δ^34^S values, indicating that they had diets dependent on prey from seagrass food webs. The nearest access to seagrass bed environments today requires longer voyages to barrier islands including the Chandeleur Islands and Cat Island (Fig. 1) (*40*). Evidence for the use of these environments is found in historical records of the collection of “ground fish” (a category including sheepshead) from the “grass and weeds” surrounding the Chandeleur Islands by at least the 1880s [e.g., (*34*), p. 576]. In the context of our isotopic evidence, it is likely that exploitation of these offshore ecosystems began much earlier, perhaps as far back as the 1820s. While it is also possible that these seagrass-dependent sheepshead may have been caught in or near seagrass beds closer to New Orleans that disappeared before being recorded, a lack of other historical evidence for these habitats suggests that the most parsimonious origin for these individuals is one of the vast seagrass habitats that are extant today.

It is also possible that these individuals were caught during annual offshore spawning congregations of sheepshead from all habitats rather than through fisheries activities directly targeting more distant seagrass habitats. A comparison of dietary and size evidence for larger post-1820 individuals, which are more likely to have been caught at new fishing grounds, provides some support for this possibility. While many of these larger individuals from the 1840–1860 spike in average sheepshead size have isotopic compositions consistent with seagrass food web–derived diets, fish with diets from a wide range of habitats are present. This could be consistent with fishing strategies that targeted spawning congregations that include comingling fish from a wider range of habitats falling outside the fishing grounds that were traditionally accessed and heavily affected in precontact and early historic periods in New Orleans. The near absence of seagrass-dependent sheepshead before the 1820s, however, along with evidence for increased catch size, suggests that wherever these specimens were caught, it was likely a new location not previously used during the precontact and early historical periods. Moreover, if, as is thought to be the case today (*22*), spawning congregations were composed of sheepshead from both nearshore and offshore populations, evidence for the apparent exclusion of individuals from seagrass ecosystems further offshore among pre-1820 fish suggests that early fisheries were not targeting spawning events.

In conjunction with historical records for demographic change (*28*), concurrent patterns in shifts between the average catch size and sheepshead habitat allow for the first detailed long-term reconstructions of how sheepshead populations responded to early fisheries impacts and the societal forces that drove them. Similarities among isotopic evidence from precontact and early 18th-century sheepshead suggest that indigenous and early French fisheries focused on the same (nearshore marine and estuarine) fishing environments, while size estimation data reveal a gradual but consistent decline in catch size through time. With an increase of immigration starting at the end of the 18th century and rapidly growing in the first half of the 1800s, New Orleans’ population soared and so too would its demand for local resources (*28*). Continued decreases in sheepshead size before the 1840s, and consistency between the isotopic compositions of sheepshead among this and earlier time periods, suggest not only that New Orleans fishers intensified their focus on sheepshead but also that these fishing activities continued to focus on the same, increasingly depleted local fishing grounds. During and after the 1820s, fishing locations expanded and began to incorporate sheepshead from populations that were not previously overfished, including fish collected from seagrass-subsidized food webs. Through the 1840 to 1860s, these new fishing grounds provided access to larger and likely more abundant sheepshead, which became increasingly important in supplying New Orleans with fish. A return to decreasing catch sizes from 1870 to 1910 indicates that the bounty of these “new” sheepshead fishing grounds was limited in the face of intensive fishing pressure.

Our findings show that sheepshead populations in the Gulf of Mexico are particularly vulnerable to overharvesting. Long before the advent of modern fishing trawlers, using only 18th- and 19th-century technologies, New Orleans fishers managed to have at least two large impacts on regional sheepshead populations. In contrast to today’s fish preferences focusing largely on drum and snapper species [e.g., (*41*)], sheepshead were highly sought-after in the past (*27*). While there are few detailed records with which to evaluate the nature and relative importance of different fish species in early historical fisheries in North America, archaeological evidence for 18th- and 19th-century fish consumption in New Orleans provides a clear indication that, more than any other taxa, sheepshead perennially remained the favored fish for a large cross-section of society (*27*). In that context, the burgeoning population and persistent popularity of sheepshead in the 18th and 19th centuries was sufficient to drive the overfishing of at least two local populations. The relatively rapid decline in sheepshead size, in one case over just one to two decades at most, suggests that, despite their highly adaptable behavior, sheepshead populations were not able to cope with sustained fishing pressure. As might be expected today, when sheepshead returns declined, 19th-century fishers expanded their fishing grounds to encompass new populations, which, in turn, saw decreasing catch size due to fishing pressure. In the broader historical context, expanding sheepshead fishing grounds was part of a suite of strategies that also included the importation of fish like snappers by railroad from the east, and which, as a whole, allowed producers to meet increasing market demand in New Orleans amidst both rising human populations and declining sheepshead stocks in the 1800s. While 19th-century records for quantifying early historical sheepshead landings in the Gulf of Mexico and New Orleans, in particular, are rare, it is likely that long-term popularity of sheepshead declined in the early 20th century, perhaps due to the increasing popularity of imported fishes like snappers, alleviating pressure on local sheepshead populations.

This sequence of events provides important signposts for managing future fisheries in the Gulf of Mexico. These results not only reveal that today’s apparent abundance of sheepshead in the region reflects a recovered (or perhaps still recovering) population but also suggest that sheepshead may require closer conservation attention. Sheepshead fisheries in the region remain comparatively unregulated, and while these fish are not the permanent focus of most fishing fleets, they are frequently taken opportunistically by commercial fishers (*22*). Today, sheepshead are rarely a target of either intensive recreational or commercial fishing efforts and are primarily sourced from bycatch, often only being kept when room permits after more valuable fish have been harvested (*22*). Sheepshead, nonetheless, share a similar textural and flavor profile with larger, more desirable fish and are sometimes masqueraded in their place. Further, sheepshead have also begun making appearances on the menus of high-end New Orleans seafood restaurants marketing sustainable fish sourcing practices, implying that sheepshead are seen by some chefs as a “safe” alternative to more heavily targeted species (*42*).

The ease with which sheepshead may be substituted for fish with higher economic value (*41*), as well as the fact that sheepshead fisheries remain broadly unregulated, leaves them vulnerable to a wide range of social and economic forces. In this context, they are particularly sensitive to shifting availability of more preferred fish. The dockside value of sheepshead, for instance, increased sharply in the 1980s when widespread regulations were enacted (*24*) to protect stocks of the more preferred red drum (*22*). This drove a spike in sheepshead landings across the Gulf of Mexico, including the first sustained direct harvesting efforts of the latter 20th century from out-of-season shrimp trawlers no longer able to pursue red drum (*22*). The apparent substitution of sheepshead for red drum serves to underscore the need to carefully consider the potential downstream impacts of regulating the harvest of high-value fish on lower value species that are likely to take their place. Like sheepshead, many of these taxa are overlooked but, as demonstrated here, can have important, if previously unrecognized, vulnerabilities to overfishing.

Results also highlight potential for contributions to addressing key fisheries and other conservation priorities in the Gulf of Mexico and beyond [e.g., (*43*, *44*)]. To our knowledge, this is the first study to explore the ecological potential for δ^34^S analyses as a proxy for reconstructing the importance of seagrass ecosystems for ancient fish communities [although see (*37*)]. As demonstrated here, the combined use of δ^13^C and δ^34^S can help to identify and quantify the importance of food webs supported by a wide variety of seagrass species, which are typically enriched in ^13^C (*45*) and depleted in ^34^S (*46*) relative to surrounding marine environments. With respect to the Gulf of Mexico, for instance, understanding the importance of submersed aquatic vegetation, like seagrasses, for sheepshead has been identified as a key management priority [(*22*), section 9.2.1]. Seagrass beds in the region are increasingly vulnerable (*47*), and the potential impact of their loss on sheepshead at different life stages is unknown. Our data provide a reference point showing that, at least in the past, seagrass beds provided important, lifelong habitats for at least some sheepshead populations. Further analyses of bones and scales from archaeological remains and archived historical and modern specimens of known age, size, and catch location could provide more detailed insights into the long-term importance of these vulnerable habitats for sheepshead at all life stages (*48*). Analyses of historical and modern sheepshead may also help assess the nature and extent of inshore and offshore population structures, another critical management priority [(*22*), section 9.1.2] that could inform improved strategies for protecting spawning aggregations at key times of the year and identify subsets of the sheepshead population that are at more risk for overfishing. Temporal variation in our dataset highlights the possibility that sheepshead caught in different areas with isotopically distinct food webs in nearshore and offshore (i.e., seagrass) regions could be identified by their isotopic composition. This opens up the possibility for using isotopic analyses to characterize the origins of fish from select areas and to assess the presence of individuals from different populations that intermingle during spring spawning events.

The results of this study also highlight future research avenues that extend beyond strictly management priorities in the Gulf of Mexico. As sheepshead are a highly adaptable species covering a range of habitats throughout the Gulf of Mexico and Atlantic Ocean, similar research could be conducted on archaeological and museum-archived assemblages from other coastal cities to map the differential historic impacts to sheepshead across a range of cultural, technological, environmental, and temporal lines. Likewise, genetic data collected from modern and ancient specimens can further address the impacts that fisheries have had on sheepshead population structure. Comparison of the mitochondrial DNA haplotypes and nuclear DNA single-nucleotide polymorphisms among specimens from different locations (e.g., Lake Pontchartrain and seagrass beds in the Chandeleur Islands) could be used to evaluate the historic population structure of sheepshead in the Gulf of Mexico. Given the reduction in genetic diversity following intensive fishing documented in other Sparid species (*10*), genetic data could also be used to document whether similar changes in genetic diversity occurred within sheepshead and whether they are tied with concomitant drops in sheepshead average size in archaeological assemblages. Similar genetic investigations of past population structure and genetic diversity have successfully been applied to other fish taxa, such as Pacific herring (*Clupea pallasii*), Chinook salmon (*Oncorhynchus tshawytscha*), and sturgeon [e.g., (*49*)]. Last, the study highlights the importance of combining stable isotope and morphological analyses in the study of past fisheries. Stable isotope analysis alone could not have detected changes in fish size (and thus overfishing), and morphological analyses could not have detected changes in fishing locations; only by combining these methods can we understand historical trends in the sheepshead fishery and the driving forces (e.g., overfishing) behind changes in fishing locations and strategies. Together, these research avenues point to the value of studying species such as sheepshead that have largely “flown under the radar” of biologists and conservationists focusing more intensively on either charismatic or more economically important species.

Despite growing calls for a better understanding of past environments to meet standards on informing conservation management practices for degraded environments (*9*, *14*), researchers of the recent past (archaeology and history) and present (ecologists) still do not routinely directly integrate perspectives between disciplinary “silos.” This represents a major obstacle, as archaeological evidence may, in many cases, offer the only source of data for addressing present and future challenges involving longer-term aspects of ecosystem rehabilitation and the sustainability of globally relevant food resources (*13*). In this context, archaeological and archived historical fish specimens hold tremendous potential for exploring long-term trends in fisheries impacts that have remained invisible to historians and fisheries researchers alike [e.g., (*11*, *50*–*52*)]. Our findings demonstrate this potential by showing that sheepshead in the Gulf of Mexico have previously been overfished on at least two occasions, highlighting the importance of considering the vulnerability of species that may be “next in line” when more desirable fish are afforded protection under regulation. More broadly, however, our findings signal a wider potential for querying the impacts of past fisheries activities when considering development of future fisheries policy.

## MATERIALS AND METHODS

### Experimental design

As bone remodels gradually over the course of an individual’s life, bone collagen isotopic compositions reflect a long-term average of dietary intake and mobility, weighted toward periods of faster growth (*53*). For this reason, isotopic variations between individuals reflect differences in diet and habitat use at the scale of entire lifetimes rather than short-term or seasonal perturbations in the isotopic composition of diet or variations in dietary behavior. Stable carbon isotope compositions (δ^13^C) of aquatic biota are governed by a highly complex set of processes including variation in carbon sources and cycling [for reviews, see (*54*, *55*)]. In the study area, key sources of variation include the extent to which local food webs rely on carbon derived from marine (higher δ^13^C) or freshwater and terrestrial (typically lower δ^13^C) sources (*56*–*58*) and nearshore (higher δ^13^C) or offshore (lower δ^13^C) production (*59*). In addition, food webs based on marine seagrasses [(*60*, *61*); in the local area: *Thalassia testudinum*, *Syringodium filiforme*, *Halodule wrightii*, and *Halophila engelmannii*] or select saltmarsh grasses [(*62*); *Spartina* spp.] are further enriched in ^13^C and will have distinctively high δ^13^C values [for review, see (*45*)]. Stable sulfur isotope compositions (δ^34^S) reflect the δ^34^S of sulfates available to primary production at the base of the food web [for review, see (*63*)]. The δ^34^S of marine environments is high [~ +20‰; (*64*)], while δ^34^S of local freshwater sources is low [~ –2‰; (*65*)]. However the high concentration of sulfur in seawater relative to local freshwater sources means that even extremely small additions of seawater [salinity > 0.6 g/liter; (*65*)] will lead to freshwater biota with marine-like δ^34^S values. Sheepshead do sometimes enter freshwater but primarily inhabit brackish and marine habitats (*66*), making it unlikely that variation in δ^34^S will reflect salinity gradients. Productivity in some regions of the study areas is, however, dominated by a variety of seagrass species and saltmarsh grasses that can incorporate sulfur from highly ^34^S-depleted sulfide sources [~ –25‰; (*67*)], resulting in biota with lower δ^34^S relative to other marine and brackish habitats [for reviews, see (*46*, *68*)]. For this reason, a primary axis for δ^34^S variation in sheepshead will be the extent to which food webs were subsidized by seagrasses and saltmarsh grasses. Although not the primary focus of this study, we also include stable nitrogen isotope compositions (δ^15^N) for sheepshead bone collagen. Unlike δ^13^C and δ^34^S, consumer δ^15^N undergoes a large stepwise ^15^N enrichment between trophic levels (*69*) and can therefore provide insights into relative trophic position. However, the highly complex nature of processes controlling nitrogen sources and cycling in aquatic environments (*70*, *71*) means that interpretations of δ^15^N in higher-level consumers like sheepshead will be challenging in the absence of baseline data from potential food sources.

### Isotopic analyses

Sheepshead bone samples (*n* = 184) were selected for isotopic analyses based on minimum number of individuals counts (e.g., using overlapping elements such as multiple left dentaries) per archaeological context where possible to minimize the chance of sampling the same individual multiple times. Samples were soaked in a 2:1 chloroform methanol solution in an ultrasonic bath (solution changed every 20 min until solution remained clear) to remove residual lipids (*72*). Samples were then demineralized in 0.5 M hydrochloric acid (HCl) and neutralized with successive rinses in type 1 water. Demineralized samples were then soaked in 0.1 M sodium hydroxide in an ultrasonic bath (solution refreshed every 20 min until solution remained clear) to remove base soluble contaminants and again rinsed in type 1 water until neutralized. Samples were then solubilized in 0.01 M HCl (pH 3) for 36 hours in an oven at 65°C and then centrifuged, and the supernatant was pipetted to a fresh tube, which was then frozen and lyophilized.

Stable carbon and nitrogen isotope analyses were performed on 0.5 mg of collagen using Elemental Analyzer (EA) 300 (Eurovector, Pavia, Italy) coupled via continuous flow to a Nu Horizon isotope ratio mass spectrometer (IRMS; Nu Horizon, Wrexham, UK) in the Water Quality Centre at Trent University (TU; Peterborough, ON, Canada) and on 1.5 mg of collagen using a Carlo Erba NA Series EA (Carlo Erba, Milan, Italy) coupled to a Thermo Delta V IRMS (Thermo Fisher Scientific, Bremen, Germany) at the Center for Applied Isotope Studies at the University of Georgia (CAIS; Athens, GA, USA). Stable sulfur isotope analyses were performed on 6.5 mg of collagen along with 10 mg of a combustion enhancer (V_2_O_5_) on a Europa ANCA EA (Europa, Crewe, UK) coupled via continuous flow to a Europa SL/20-20 IRMS at Iso-Analytical (IA; Cheshire, UK). At TU, sample isotopic compositions were calibrated using a two-point curve [anchored to USGS (U.S. Geological Survey)-40 and USGS-41a; (*73*, *74*)] for δ^13^C and δ^15^N relative to VPDB (Vienna Pee Dee Belemnite) and AIR (Ambient Inhalable Reservoir), respectively. At CAIS, sample isotopic compositions were calibrated using a two-point curve (anchored to internal standards “Spinach” and “1577C”). At IA, isotopic compositions were calibrated using a three-point curve [anchored to internal standards IA-R061, IA-R025, and IA-R026, themselves anchored to IAEA (International Atomic Energy Agency)-S-1 and NBS (National Bureau of Standards) 127; (*75*)] for δ^34^S relative to VCDT (Vienna-Canyon Diablo Troilite). Known (calibration standards) and long-term observed averages (check standards) for all analytical reference materials are reported in table S3. Averages and SD for calibration standards (table S4), check standards (table S5), and sample replicates (data S4) for all analytical sessions are also available in the Supplementary Materials. For δ^34^S, δ^13^C, and δ^15^N, systematic errors [*u*_(bias)_] were ±0.11‰, ±0.07‰, and ±0.20‰, respectively; random errors [*uR*_(*w*)_] were ±0.24‰, ±0.05‰, and ±0.19‰, respectively; and standard uncertainty was ±0.33‰, ±0.13‰, and ±0.28‰, respectively (*76*). Collagen quality control (QC) was assessed using C:N (2.9 to 3.6), %C (>13%), and %N (>4.8%) criteria (*77*).

### Zooarchaeology

Osteological analyses were undertaken in the University of New Orleans’ (UNO) Archaeology Laboratory following standard zooarchaeological procedures (*78*). Complete fish bone assemblages from six archaeological sites with components spanning the past 2500 years formed the basis of these analyses. Initial identification of all sheepshead specimens in these assemblages was accomplished through direct comparison with fish skeletons in the UNO comparative fish skeletal collection, which includes examples of all fish species commonly found at archaeological sites in the study area. Size estimation for the sheepshead specimens from these assemblages was accomplished through comparison with a catalog of modern sheepshead skeletons spanning the complete range of sizes identified in the archaeological record. A total of 353 archaeological sheepshead specimens were complete enough to use for size estimation, and they were compared to the modern skeletal specimen catalog and assigned an estimated standard length value based on the modern specimen that they matched most closely in size. Although archaeological size estimation projects often use regression formulae to model the relationship of fish length and specific bone measures (e.g., width of vertebral centra and maximal height of the proximal dentary), size matching by direct comparison with modern specimens of known sizes allows analysts to more readily provide estimates for a wider range of archaeological specimens, especially fragmented skeletal elements lacking the landmarks chosen for regression formulae, and thereby incorporate a wider range of archaeological specimens into size estimation datasets.

### Statistical analyses

Temporal trends in zooarchaeological data were compared between group means using PAST (PAleontological STatistics) Version 3.22 (*79*). We assessed normality of distribution with Shapiro-Wilk tests. When one or more groups were not normally distributed, a Mann-Whitney *U* test was used for comparisons. When variances were determined to be equal, we used a Student’s *t* test to compare two normally distributed groups. We assessed homogeneity of variance using a Levene’s test. For testing the significance of correlations between δ^13^C and δ^34^S, we used a Peason’s *r* test. Isotopic variation was quantified using the standard bivariate ellipse corrected for sample size (SEAc) and total area (TA; also known as convex hull area) with the SIBER package in R 3.0.3 (*80*).

## SUPPLEMENTARY MATERIALS

Supplementary material for this article is available at http://advances.sciencemag.org/cgi/content/full/7/32/eabh2525/DC1
